# Testing active choice for screening practitioner’s gender in endoscopy among disinclined women: An online experiment

**DOI:** 10.1177/0969141318806322

**Published:** 2018-11-14

**Authors:** Sandro T Stoffel, Yasemin Hirst, Alex Ghanouni, Lesley M McGregor, Robert Kerrison, Wouter Verstraete, Ailish Gallagher, Jo Waller, Christian von Wagner

**Affiliations:** Research Department of Behavioural Science and Health, University College London, London, UK

**Keywords:** Decision making, cancer screening, online experiment, gender preferences, endoscopist gender, attitude change

## Abstract

**Objectives:**

A large proportion of women have a preference for a same-gender endoscopy practitioner. We tested how information about practitioner gender affected intention to have bowel scope screening in a sample of women disinclined to have the test.

**Methods:**

In an online experimental survey, women aged 35–54 living in England who did not intend to participate in bowel scope screening (N = 1060) were randomised to one of four experimental conditions: (1) *control* (practitioner’s gender is unknown), (2) *opposite-gender* (male practitioner by default), (3) *same gender* (female practitioner by default), and (4) *active choice* (the patient could choose the gender of the practitioner). Intention was measured following the interventions.

**Results:**

Of 1010 (95.3%) women who completed the survey, most were White-British (83.6%), and working (63.3%). Compared with *control*, both *active choice* and *same-gender* conditions increased intention among disinclined women (9.3% vs. 16.0% and 17.9%; OR: 1.85; 95% CI: 1.07–3.20 and OR: 2.07; 95% CI: 1.23–3.50). There were no differences in intention between the *opposite-gender* and *control* conditions (9.8% vs. 9.3%; OR: 1.06; 95% CI: 0.60–1.90) or the *active choice* and *same-gender* conditions (16.0% vs. 17.9%: OR: 0.89; 95% CI: 0.55–1.46, using *same gender* as baseline).

**Conclusions:**

Offering disinclined women a same-gender practitioner, either by choice or default, increased subsequent intention, while an opposite gender default did not negatively affect intention. Reducing uncertainty about gender of practitioner could positively affect uptake in women, and should be tested in a randomised controlled trial.

## Introduction

Colorectal cancer is one of the most common causes of cancer death.^1,2^ Incidence can be reduced using an endoscopic flexible sigmoidoscopy test (bowel scope), to detect and remove pre-cancerous adenomatous polyps in the bowel.^3^ The English Bowel Cancer Screening Programme offers bowel scope screening (BSS) as a one-off test to men and women aged 55. BSS involves considerable individual costs, including taking time off work, undergoing an enema-based preparation, and the potential embarrassment and pain caused by the test itself. This may explain why the test uptake is considerably lower than that for the home-based stool test introduced in 2006 (43% vs. 54).^4,5^ There is also a contrast in the relationship between uptake and gender. Women are more likely to take part in screening via the stool test than men (56% vs. 51%),^5^ but less likely than men to participate in BSS (42% vs. 45%).^4^ This finding indicates that women may experience unique barriers to screening using the BSS test.^6^ One potential barrier is embarrassment related to the gender of the practitioner.^7,8^

In the absence of experimental studies manipulating the practitioner’s gender, the effect of offering choice on screening behaviour as well as the effect of endoscopist’s gender *per se* remain uncertain. Previous studies have shown that women are more likely to have a preference for a female practitioner, and to be more willing to participate, if the practitioner is female.^7,9^ This hypothesis was tested in a recent feasibility study in England, which re-invited previous BSS non-attenders for another appointment, and offered a choice of practitioner gender. In this study, female non-attenders unanimously chose a same-gender practitioner.^10^ There is also evidence that women are more willing to wait for a female practitioner. For example, in a US study, 45% of female patients expressed a preference for a female practitioner, compared with only 4% of men expressing a preference. Of these women, 90% indicated that they would be willing to wait up to one month for an practitioner of the preferred gender.^11^ A UK study also found that 34% of women were willing to wait up to one month to have a female practitioner.^9^ Despite this strong observational evidence, there is a lack of evidence from randomised controlled trials comparing screening intentions or uptake in people with or without the choice of practitioner’s gender.

This study was designed to test how information about the gender of the practitioner influences intention to participate in the screening programme among previously disinclined women. Women were presented with information about the test that variously stated that the practitioner’s gender would be unknown, certainly male, certainly female, or that participants would be able to choose the gender of the practitioner before the appointment. We hypothesised that non-intending women would be more willing to participate in BSS if the gender of the practitioner was female by default or if they could choose the gender of the practitioner, compared with the current standard (gender of the practitioner is unknown) or a male practitioner by default. We investigated the extent to which women would prefer being able to choose the sex of their practitioner or be offered a female by default. As both UK and US studies show that not all women prefer a female practitioner (up to 5% of women prefer a male practitioner), we hypothesised that allowing participants to choose the gender of their practitioner would lead to a small additional improvement over and above offering a same-gender practitioner as default.^8,9,11^

## Methods

In 2018, women aged 35–54 living in the UK were invited to take part in an online survey. Only those who gave explicit written consent for their data to be used and published as part of this research project continued in the survey. Study participants were presented with a short description of BSS (see Supplemental material) and were asked to correctly answer a compulsory comprehension question by identifying BSS as a test that involves inserting a flexible tube into the back passage. Participants were then asked to indicate their intentions to participate in the screening test using the question ‘Would you take up the offer if you were invited to have the bowel scope screening test?’ with responses on a fully labelled four-point scale (‘definitely not’, ‘probably not’, ‘probably yes’ and ‘definitely yes’). Those who had indicated that they would probably or definitely do the test, were redirected to the study briefing and final survey page where they were thanked for their participation. Those who stated that they would definitely or probably not do the screening test were individually randomised to one of four experimental conditions with different information about the gender of the practitioner: (1) *control* (‘When you are invited, you are not given any information about the gender of the person who will do the test’); (2) *opposite gender* (‘When you are invited, you are told that the person who will do the test is a man’); (3) *same gender* (‘When you are invited, you are told that the person who will do the test is a woman’); and (4) *active choice* (‘When you are invited, you will be able to choose the gender of the person who will do the test’). The allocation ratio for the four parallel conditions was 1:4. After receiving information about the screening procedure, respondents had a second manipulation check that required them to correctly repeat the information about the gender of the practitioner before completing the rest of the survey.

The primary outcome was intention to attend BSS screening. This was measured using the same-intention question as described above. The secondary outcomes focussed on the impact of the gender of the practitioner on possible BSS barriers and on the preferred gender of the practitioner. To explore if the gender of the practitioner had an impact on other possible BSS barriers, we included a question on embarrassment (‘I think having the bowel scope screening test would make me feel embarrassed’), pain (‘I think having the bowel scope screening test would be painful’), exposure (‘I think having the bowel scope screening test would make me feel exposed’), comfort (‘I think having the bowel scope screening test would be comfortable’), appeal (‘I think having the bowel scope screening test would be appealing’), and how off-putting the test was seen as being (‘I think having the bowel scope screening test would be off-putting’). All items were measured on fully-labelled five-point Likert scales (‘strongly disagree’, ‘disagree’, ‘neither agree nor disagree’, ‘agree’, and ‘strongly agree’). Participants in the active choice condition were also asked about what gender of practitioner they would prefer (‘When you are asked to choose the gender of the person doing the test, what will you choose?’) with three possible response options: ‘a man’, ‘a woman’, or ‘don’t mind’. All primary and secondary outcomes were measured immediately after the interventions.

Respondents’ sociodemographic characteristics such as age, ethnicity, marital status, education, employment, car and house ownership, and health status were also collected. We used the sociodemographic variables as covariates in multivariable logistic regressions to investigate the effect of the experimental conditions on dichotomised intention to take part (‘probably yes’ and ‘definitely yes’ vs. ‘probably not’ and ‘definitely not’) and perception of the BSS (‘agree’ and ‘strongly agree’ vs. ‘neither agree nor disagree’, ‘disagree’ and ‘strongly disagree’). The reclassification of the outcome variables was due to low frequencies in some answer categories.

A sample size calculation indicated that the minimum number of individuals per group was 250 in order to detect a difference of at least 10% in proportion of non-intenders, with a power of 80% at the 5% level of significance. We used a Chi-square test of independence and multivariable logistic regression adjusting for initial intention and sociodemographic variables to investigate the effect of the conditions on screening intentions and perception of the screening test. While we only report odds ratios (ORs) for the experimental manipulation in the text, the full models showing all the covariates are displayed in Supplemental Tables 2 and 3. The statistical analysis was conducted with Stata/SE version 15.1 (StataCorp LP, College Station, TX).

This research project was approved by the UCL Research Ethics Committee (approval number 13439/001).

## Results

**Figure 1.**
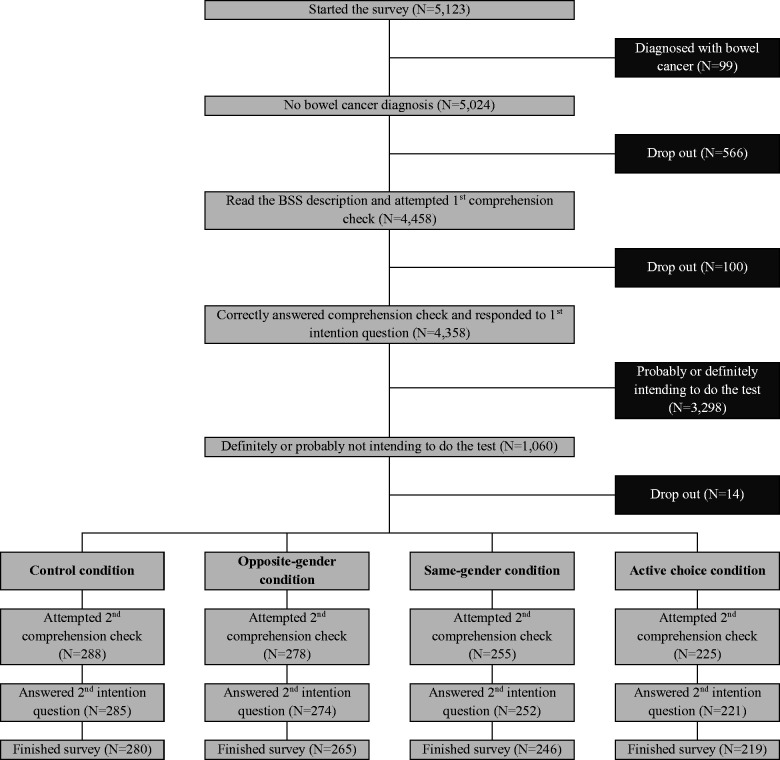
Study flow chart.

Out of 5123 women aged 35–54, living in England, and registered on a survey panel (Survey Sampling International) who were invited to complete an online survey on bowel cancer screening, 99 (1.9%) were immediately excluded because of a self-reported bowel cancer diagnosis. The remaining 5024 (98.1%) read the short description of BSS and were asked to complete a comprehension check. Of the 666 participants who dropped out of the survey at that point, 566 (85.0%) did it before and 100 (15.0%) after attempting the comprehension check at least once (Figure 1). In total, 4358 (85.1%) of those invited read the description, correctly, answered the comprehension check, and stated their intention. Of these responders, 1060 (24.3%) stated that they would definitely (248, 23.4%) or probably (812, 76.6%) not do the screening test, and were randomised to one of four experimental conditions: 288 were randomly allocated to the control condition, 278 to the opposite-gender condition, 255 to the same-gender condition, and 225 to the active choice condition. Across conditions, 50 (4.7%) did not finish the survey after the randomisation, leaving a final sample of 1010, who were all included in the analysis: 280 participants in the control condition, 265 in the opposite-gender condition, 246 in the same-gender condition, and 219 in the active choice condition. Most participants were aged between 35 and 44 (59.4%), had paid work (63.3%), were married or cohabiting (62.4%), White-British (83.8%), owned a car (51.4%), were non-homeowners (49.3%), did not have a university degree (76.0%), reported good or excellent health (54.0%), and stated in the first intention question (77.4%) that they would probably not undergo screening. There were no statistically significant differences in sociodemographic characteristics and initial intention, indicating that there were no imbalances due to levels of drop-out varying among the four experimental conditions (see Supplemental Table 1).

**Figure 2.**
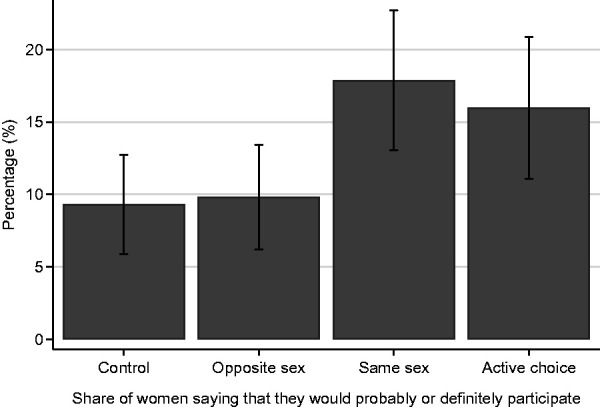
Proportion of women stating that they would probably or definitely participate (post-manipulation).

Non-intenders were more likely to become intenders if they had been randomised to the same-gender or active choice condition compared with the opposite-gender or the control condition (17.9% and 16.0% vs. 9.3% and 9.8% respectively) (see Figure 2 and Table 1). The adjusted logistic regression in Table 2 confirmed that, compared with *control*, both *same gender* and *active choice* significantly increased screening intention (17.9% vs. 9.3%; OR 2.107; 95% CI: 1.23–3.50, *p* = 0.006 and 16.0% vs. 9.3%: OR 1.85; 95% CI: 1.07–3.20, *p* = 0.027). Informing women that their practitioner would be male did not affect intention (9.8% vs. 9.3%; OR 1.06; 95% CI: 0.60–1.90, *p* = 0.837). Using the *same-gender condition* as baseline in the regression revealed that there was no statistically significant difference between the *same-gender* and *active choice* conditions (17.9% vs. 16.0%; OR 0.89; 95% CI: 0.55–1.46, *p* = 0.651). An examination of gender preferences among women in the *active choice* condition revealed that, although most women stated that they would prefer a female practitioner (N = 141, 64.4%), a significant proportion of them had no preference at all (N = 73, 33.3%), and only a few would request a male practitioner (N = 5, 2.3%). Although our study was not designed to compare those who were allocated male and female practitioners with *active choice*, our data suggest no statistically significant difference (16.0% vs. 13.7%, *x*^2^ (3, N = 730) = 0.649, *p* = 0.421), although our study lacked statistical power to make meaningful comparisons between the *active choice* condition and the *same-gender* and *opposite-gender* condition combined.

**Table 1.** Effect on dichotomised intentions and perception of the screening test.

	Control(N = 280)	Opposite gender(N = 265)	Same gender(N = 246)	Active choice(N = 219)	Overall	*p* value*
Intentions after exposure						
Def/prob not	254 (90.7%)	239 (90.2%)	202 (82.1%)	184 (84.0%)	879 (87.0%)	0.005
Prob/def yes	26 (9.3%)	26 (9.8%)	44 (17.9%)	35 (16.0%)	131 (13.0%)	
Expecting test to be embarrassing						
Disagree	83 (29.6%)	65 (24.5%)	75 (30.5%)	73 (33.3%)	296 (29.3%)	0.186
Agree	197 (70.4%)	200 (75.5%)	171 (69.5%)	146 (66.7%)	714 (70.7%)	
Expecting test to be painful						
Disagree	113 (40.4%)	94 (35.5%)	101 (41.1%)	94 (42.9%)	402 (39.8%)	0.367
Agree	167 (59.6%)	171 (64.5%)	145 (58.9%)	125 (57.1%)	608 (60.2%)	
Expecting test to be exposing						
Disagree	92 (32.9%)	68 (25.7%)	75 (30.5%)	78 (35.6%)	313 (31.0%)	0.102
Agree	188 (67.1%)	197 (74.3%)	171 (69.5%)	141 (64.4%)	697 (69.0%)	
Expecting test to be comfortable						
Disagree	258 (92.1%)	240 (90.6%)	231 (93.9%)	200 (91.3%)	929 (92.0%)	0.554
Agree	22 (7.9%)	25 (9.4%)	15 (6.1%)	19 (8.7%)	81 (8.0%)	
Expecting test to be appealing						
Disagree	251 (89.6%)	252 (95.1%)	230 (93.5%)	203 (92.7%)	936 (92.7%)	0.097
Agree	29 (10.4%)	13 (4.9%)	16 (6.5%)	16 (7.3%)	74 (7.3%)	
Expecting test to be off-putting						
Disagree	88 (31.4%)	80 (30.2%)	83 (33.7%)	79 (36.1%)	330 (32.7%)	0.528
Agree	192 (68.6%)	185 (69.8%)	163 (66.3%)	140 (63.9%)	680 (67.3%)	

*p value refers to Chi-square test of independence.

**Table 2.** Unadjusted and adjusted regressions on post-exposure dichotomised screening intentions.

	Unadjusted model		Adjusted model^a^	
	OR	95% CI	OR	95% CI
Condition				
Control	Ref.		Ref.	
Opposite gender	1.063	0.600–1.882	1.062	0.597–1.889
Same gender	2.128	1.267–3.575**	2.074	1.230–3.496**
Active choice	1.858	1.081–3.194*	1.851	1.073–3.195*
*N*	1010		1010	
*R*^2^ *(Nagelkerke)*	0.023		0.039	

^a^Model is adjusted for initial intentions, age, ethnicity, marital status, education, car and house ownership, work and health status.

*p < 0.05; **p < 0.01.

A majority of respondents agreed with the statement that the test would be embarrassing (70.7%), painful (60.2%), exposing (69.0%), and off-putting (67.3%). Only a small group of respondents expected the test to be comfortable (8.0%) or found it appealing (7.3%). However, the adjusted logistic regressions reported in Table 3 and the *p* values of the Chi-square tests reported in Table 1 did not indicate that communicating the practitioner’s gender or offering an active choice significantly affected the perception of the screening programme. The only statistical significant difference was that women who were told that their practitioner would be male were less likely to perceive the test as appealing (4.9% vs. 10.4%; OR 0.46; 95% CI: 0.23–0.91, *p =* 0.026).

**Table 3.** Adjusted regressions on post-exposure dichotomised perception of the screening test.

	Embarrassing		Painful		Exposing		Comfortable		Appealing		Off-putting	
	OR	95% CI	OR	95% CI	OR	95% CI	OR	95% CI	OR	95% CI	OR	95% CI
Condition												
Control	Ref.		Ref.		Ref.		Ref.		Ref.		Ref.	
Opposite gender	1.285	0.873–1.892	1.286	0.904–1.829	1.411	0.965–2.062	1.326	0.721–2.441	0.459	0.231–0.910*	1.043	0.720–1.510
Same gender	0.966	0.660–1.413	0.974	0.684–1.387	1.132	0.777–1.649	0.771	0.388–1.533	0.608	0.320–1.155	0.918	0.633–1.331
Active choice	0.847	0.575–1.249	0.915	0.636–1.316	0.894	0.611–1.307	1.144	0.597–2.191	0.699	0.367–1.332	0.807	0.552–1.179
*N*	1010		1010		1010		1010		1010		1010	

All models are adjusted for initial intentions, age, ethnicity, marital status, education, car and house ownership, work and health status.

*p < 0.05; **p < 0.01.

## Discussion

This is the first study to have investigated how the communication of the practitioner’s gender affects screening intentions among disinclined women. We found that offering disinclined women a same-gender practitioner, either by choice or default, significantly increased subsequent screening intention. This suggests that the opportunity to have a same-gender practitioner during BSS could make an important contribution to reducing women’s concerns and facilitating participation. We did not find any evidence that offering an active choice would increase screening intentions over and above allocating women female practitioners by default, as almost no women in our study expressed a preference for a male practitioner. Thus, offering prospective invitees the choice of practitioner’s gender compared with simply allocating a female practitioner only benefited a few women. For those indicating ‘no preference’, offering an irrelevant choice between male and female practitioner may have caused choice overload, and subsequently put them off attending BSS.^12^ Previous research suggests that if women are offered a same-gender practitioner, their anticipated embarrassment may be reduced;^8^ however, our study did not find any evidence that offering disinclined women a female practitioner instead of a male one reduced their expected feeling of embarrassment or exposure. This may suggest that the practitioner’s gender does not influence the perceived level of these feelings, but rather the women’s capability to cope with them.

One important finding of our study was that each of our experimental conditions including a not tested combination of both default conditions (i.e. random allocation of male and female practitioners), led to significantly higher screening intentions than control. While the study was underpowered to detect differences between the active choice and same-gender conditions, we can be confident that reducing uncertainty of the practitioner’s gender is likely to affect overall uptake positively. Given that the *opposite-gender* condition did not lead to fewer women intending to take part compared with the control condition (no information given) also provided reassurance that increased transparency of the gender of the practitioner would not have detrimental effects, even among those women who would be explicitly told their practitioner was male.

These findings are particularly important as offering a same-gender practitioner may not always be feasible. For same-gender practitioners to become a viable option across all centres, a considerable increase in the female endoscopic workforce would be required.^13^ In the meantime, eliciting preferences, or even just informing participants of the practitioner’s gender, would be considerably more feasible and likely to have a positive uptake effect.

This study has some important limitations, which call for follow-up research. The current sample consisted of women aged 35 to 54 who were not screening eligible at the time they answered the survey, so the screening scenario may not have been relevant for them. Further experiments need to test whether these findings also hold for women who are screening eligible. Our decision to use non-eligible women was motivated by the logistical difficulties of recruiting a large sample of women aged exactly 55. In addition, while we believe that our online experiment has a high level of internal validity due to the compulsory manipulation checks on the screening test and the experimental manipulation, we measured screening intentions in a hypothetical scenario, and not real behaviour. Literature on the intention-behaviour gap suggests that changing intentions may not necessary lead to a change in behaviour.^14^ Also, as the study was hypothetical, we were also not able to measure the potential impact of these conditions on patient-reported experience of the test. Both of these aspects could be tested in a follow-up randomised controlled trial. This experimental study focused on non-intenders, but to check the overall effect of the practitioner’s gender on screening behaviour, future research might usefully also include intenders. Finally, the online experiment was set in the context of BSS, which is currently only offered in England. However, the findings have important implications beyond primary screening and would also apply to women receiving colonoscopy to follow up an abnormal screening result, or in the diagnostic/surveillance context.

## Conclusions

This online study demonstrated the importance of a practitioner’s gender on the intention of disinclined women to accept an invitation to have BSS. Informing women that they can choose the practitioner’s gender, or have a female practitioner by default, increased subsequent intention relative to control. The potential of providing information about the gender of practitioner for improving BSS uptake in women should be tested in a randomised controlled trial.

## Supplemental Material

Supplemental material for Testing active choice for screening practitioner’s gender in endoscopy among disinclined women: An online experiment Supplemental Material for Testing active choice for screening practitioner’s gender in endoscopy among disinclined women: An online experiment by Sandro T Stoffel, Yasemin Hirst, Alex Ghanouni, Lesley M McGregor, Robert Kerrison, Wouter Verstraete, Ailish Gallagher, Jo Waller and Christian von Wagner in Journal of Medical Screening

## Author's note

While the design and analysis plans for the experiment was not preregistered, all data and materials are publicly available via the Open Science Framework and can be accessed at https://osf.io/s84fh/.
