# Supplementary material for: Testing active choice for screening practitioner’s gender in endoscopy among disinclined women: An online experiment
Source: J Med Screen. 2018 Nov 14;26(2):98–103. doi: 10.1177/0969141318806322 (PMC6484820; doi:10.1177/0969141318806322)
Supplement: Supplemental material for Testing active choice for screening practitioner’s gender in endoscopy among disinclined women: An online experiment [file Supplemental_Material.pdf]

Appendix: Addressing women's barriers to bowel scope screening

Table 1 Descriptive statistics of the study population (N=1,010)

	Control (N=280)		Opposite gender (N=265)		Same gender (N=246)		Active choice (N=219)		Overall		p-value*
**Age**											
35-44	179	(63.9%)	151	(57.0%)	147	(59.8%)	123	(56.2%)	600	(59.4%)	0.648
45-54	101	(36.1%)	114	(43.0%)	99	(40.2%)	96	(43.8%)	410	(40.6%)	
**Living status**											
Married/cohab.	166	(59.3%)	170	(64.2%)	155	(63.0%)	139	(63.5%)	630	(62.4%)	0.381
Single/div./wid.†	114	(40.7%)	95	(35.8%)	91	(37.0%)	80	(36.5%)	380	(37.6%)	
**Ethnicity**											
White British	233	(83.2%)	226	(85.3%)	203	(82.5%)	184	(84.0%)	846	(83.8%)	0.430
Other	47	(16.8%)	39	(14.7%)	43	(17.5%)	35	(16.0%)	164	(16.2%)	
**Education**											
No uni .degree	211	(75.4%)	194	(73.2%)	190	(77.2%)	173	(79.0%)	768	(76.0%)	0.479
Uni. degree	69	(24.6%)	71	(26.8%)	56	(22.8%)	46	(21.0%)	242	(24.0%)	
**Paid work**											
No	110	(39.3%)	103	(38.9%)	81	(32.9%)	77	(35.2%)	371	(36.7%)	0.617
Yes	170	(60.7%)	162	(61.1%)	165	(67.1%)	142	(64.8%)	639	(63.3%)	
**Car ownership**											
No	82	(29.3%)	81	(30.6%)	63	(25.6%)	56	(25.6%)	282	(27.9%)	0.487
Yes	198	(70.7%)	184	(69.4%)	183	(74.4%)	163	(74.4%)	728	(72.1%)	
**House ownership**											
No	146	(52.1%)	128	(48.3%)	130	(52.8%)	108	(49.3%)	512	(50.7%)	0.691
Yes	134	(47.9%)	137	(51.7%)	116	(47.2%)	111	(50.7%)	498	(49.3%)	
**Self-reported health status**											
Good/excellent	144	(51.4%)	152	(57.4%)	127	(51.6%)	122	(55.7%)	545	(54.0%)	0.669
Poor/fair	136	(48.6%)	113	(42.6%)	119	(48.4%)	97	(44.3%)	465	(46.0%)	
**Intentions before exposure**											
Definitely not	70	(25.0%)	56	(21.1%)	52	(21.1%)	50	(22.8%)	228	(22.6%)	0.669
Probably not	210	(75.0%)	209	(78.9%)	194	(78.9%)	169	(77.2%)	782	(77.4%)	

*p-value refers to Chi-Square test of independence

†Single , divorced or widow

Table 2 Adjusted logistic regression model on dichotomised screening intentions

	OR	95% CI
**Condition**		
Control	Ref.	
Opposite gender	1.062	0.597 - 1.889
Same gender	2.074	1.230 - 3.496**
Active choice	1.851	1.073 - 3.195*
**Initial intention**		
Definitely not	Ref.	
Probably not	1.279	0.792 - 2.064
**Age**		
35-44	Ref.	
45-54	1.060	0.723 - 1.553
**Living status**		
Married/cohab	Ref.	
Single/div./wid.	1.007	0.666 - 1.523
**Ethnicity**		
White	Ref.	
Other	0.871	0.510 - 1.488
**Education**		
No uni .degree	Ref.	
Uni. degree	1.312	0.855 - 2.016
**Paid employment**		
No	Ref.	
Yes	1.343	0.881 - 2.045
**Car ownership**		
No	Ref.	
Yes	1.322	0.814 - 2.147
**House ownership**		
No	Ref.	
Yes	1.043	0.696 - 1.562
**Self-reported health status**		
Good/excellent	Ref.	
Poor/fair	1.271	0.861 - 1.875
R^2 *(Nagelkerke)*	0.039	

(* p<0.05; ** p<0.01)

Table 3 Adjusted regressions on post-exposure dichotomised perception of the screening test

	Embarrassing		Painful		Exposing		Comfortable		Appealing		Off-putting	
	OR	95% CI	OR	95% CI	OR	95% CI	OR	95% CI	OR	95% CI	OR	95% CI
**Condition**												
Control	Ref.		Ref.		Ref.		Ref.		Ref.		Ref.	
Opposite gender	1.285	0.873 - 1.892	1.286	0.904 - 1.829	1.411	0.965 - 2.062	1.326	0.721 - 2.441	0.459	0.231 - 0.910*	1.043	0.720 - 1.510
Same gender	0.966	0.660 - 1.413	0.974	0.684 - 1.387	1.132	0.777 - 1.649	0.771	0.388 - 1.533	0.608	0.320 - 1.155	0.918	0.633 - 1.331
Active choice	0.847	0.575 - 1.249	0.915	0.636 - 1.316	0.894	0.611 - 1.307	1.144	0.597 - 2.191	0.699	0.367 - 1.332	0.807	0.552 - 1.179
**Initial intentions**												
Definitely not												
Probably not	1.117	0.804 - 1.551	0.760	0.556 - 1.040	0.985	0.709 - 1.369	0.536	0.325 - 0.885*	0.580	0.344 - 0.979*	0.876	0.632 - 1.214
**Age**												
35-44	Ref.		Ref.		Ref.		Ref.		Ref.		Ref.	
45-54	0.760	0.572 - 1.011	0.732	0.562 - 0.953*	0.857	0.646 - 1.136	0.582	0.350 - 0.967*	0.696	0.415 - 1.165	0.969	0.734 - 1.278
**Living status**												
Married/cohab	Ref.		Ref.		Ref.		Ref.		Ref.		Ref.	
Single/div./wid.	0.778	0.574 - 1.055	0.861	0.648 - 1.144	0.747	0.552 - 1.009	1.033	0.616 - 1.730	1.090	0.642 - 1.851	0.954	0.709 - 1.284
**Ethnicity**												
White	Ref.		Ref.		Ref.		Ref.		Ref.		Ref.	
Other	0.555	0.387 - 0.796**	0.920	0.646 - 1.309	0.640	0.446 - 0.920*	0.950	0.510 - 1.770	0.834	0.430 - 1.617	0.547	0.385 - 0.778**
**Education**												
No uni .degree	Ref.		Ref.		Ref.		Ref.		Ref.		Ref.	
Uni. degree	1.400	0.992 - 1.974	1.032	0.758 - 1.404	1.596	1.132 - 2.251**	0.592	0.321 - 1.094	1.137	0.658 - 1.966	1.054	0.764 - 1.454
**Paid work**												
No	Ref.		Ref.		Ref.		Ref.		Ref.		Ref.	
Yes	0.974	0.721 - 1.316	0.994	0.751 - 1.314	1.012	0.753 - 1.362	0.940	0.572 - 1.544	1.601	0.913 - 2.807	0.803	0.598 - 1.077
**Car ownership**												
No	Ref.		Ref.		Ref.		Ref.		Ref.		Ref.	
Yes	1.156	0.826 - 1.618	1.510	1.102 - 2.068*	1.014	0.726 - 1.415	1.520	0.838 - 2.757	1.088	0.592 - 2.003	1.064	0.767 - 1.476
**House ownership**												
No	Ref.		Ref.		Ref.		Ref.		Ref.		Ref.	
Yes	1.264	0.934 - 1.711	0.988	0.746 - 1.308	1.510	1.120 - 2.035**	0.746	0.451 - 1.233	0.993	0.586 - 1.681	1.463	1.092 - 1.960*
**Self-reported health status**												
Good/excellent	Ref.		Ref.		Ref.		Ref.		Ref.		Ref.	
Poor/fair	1.203	0.899 - 1.611	1.237	0.945 - 1.621	1.456	1.090 - 1.946*	0.735	0.450 - 1.202	0.856	0.516 - 1.423	1.106	0.835 - 1.465
N	1,010		1,010		1,010		1,010		1,010		1,010	
R2 (Nagelkerke)	0.046		0.030		0.054		0.045		0.040		0.036	

(* p<0.05; ** p<0.01)
